# Albumin to Globulin ratio, Neutrophil to Lymphocyte ratio, and Globulin levels do not outperform ESR or CRP when diagnosing periprosthetic joint infection

**DOI:** 10.1186/s12891-022-05357-y

**Published:** 2022-04-30

**Authors:** Jing-bo Jiao, Jin-cheng Huang, Xiao Chen, Yi Jin

**Affiliations:** 1 Department of Orthopaedics, Henan University People’s Hospital, Henan Provincial People’s Hospital, Zhengzhou, Henan China; 2 Department of Orthopaedics, Henan Provincial People’s Hospital, Henan University People’s Hospital, Zhengzhou University People’s Hospital, Zhengzhou, Henan China

**Keywords:** Globulin, Albumin to Globulin Ratio, Neutrophil to Lymphocyte Rate, Diagnosis, Periprosthetic joint infection

## Abstract

**Objective:**

To evaluate the relative performance of clinical readouts including serum C-reactive protein (CRP) levels, the erythrocyte sedimentation rate (ESR), globulin (GLB) levels, the albumin to GLB ratio (A/G), and the neutrophil to lymphocyte ratio (NLR) when diagnosing periprosthetic joint infection (PJI).

**Methods:**

Clinical data was collected from 115 individuals diagnosed in our department between January 2017 and December 2020 with either chronic PJI (29 female, 24 male; median age 71.00 years [range, 41–94 years]) or aseptic loosening (30 female, 32 male; median age 68.50 years [range, 34–85 years]). Patient demographic data were compared, and the relative sensitivity and specificity of preoperative GLB, ESR, CRP, NLR, and A/G values as predictors of PJI diagnosis were assessed.

**Results:**

Median globulin levels in the PJI and aseptic groups were 31.700 g/L (interquartile range [IQR], 28.400—35.300) and 26.600 g/L (IQR, 24.375—30.550), respectively (*p* < 0.001). The median A/G values in the PJI and aseptic groups were 1.150 (IQR, 0.960—1.255) and 1.510 (IQR, 1.265—1.670), respectively (*p* < 0.001). The median NLR values in the PJI and aseptic groups were 2.510 (IQR, 1.900—3.335) and 1.850 (IQR, 1.425 to 2.362), respectively (*p* < 0.001). The median ESR values in the PJI and aseptic groups were 53.000 mm/h (IQR, 35.000—76.500) and 16.000 mm/h (IQR, 7.000—33.000), respectively (*p* < 0.001). Median CRP levels in the PJI and aseptic groups were 24.890 mg/L (IQR, 10.595—54.095) and 2.245 mg/L (IQR, 0.865—8.6075), respectively (*p* < 0.001). Area under the receiver operating characteristic (ROC) curve (AUC) values for CRP, ESR, GLB, A/G, and NLR were 0.841 (95% confidence interval, 0.761–0.903), 0.850 (0.771–0.910), 0.747 (0.658–0.824), 0.779 (0.692–0.851), and 0.708 (0.616–0.789), respectively. When GLB > 26.6 g/L, A/G < 1.32, and NLR > 2.1 were utilized as threshold values to diagnose PJI, GLB and A/G were found to exhibit superior sensitivity (90.57%, 81.13%) to that observed for CRP (71.70%) and ESR (79.25%), but the specificity of these two metrics (GLB: 51.61%, A/G: 72.58%) was significantly reduced relative to that for CRP (87.10%) or ESR (75.81%). ROC analyses further revealed that NLR did not exhibit significant advantages in sensitivity (73.58%) or specificity (70.97%) relative to CRP or ESR.

**Conclusion:**

Globulin levels, NLR values, and A/G values do not outperform ESR or CRP levels when used to diagnose PJI.

**Supplementary Information:**

The online version contains supplementary material available at 10.1186/s12891-022-05357-y.

## Introduction

Total knee/hip arthroplasty (TKA and THA) are increasingly frequently employed and highly effective treatments for patients suffering from end-stage joint conditions including femoral head necrosis and knee osteoarthritis, restoring joint functionality while alleviating pain in affected patients. However, as these procedures continue to become more widespread, postoperative complication rates continue to rise, with periprosthetic joint infection (PJI) being a particularly common and dangerous such complication [1–3]. Debilitating PJI is estimated to affect 1–2.5% of patients following TJA, imposing a substantial burden on patients and the healthcare system as a whole [4].

Early symptoms of PJI can be inconsistent, and diagnosing the condition using traditional serological indicators may be ineffective. There is thus a critical need to identify reliable and easily measured serological factors that can guide the diagnosis of these infections in order to aid in timely treatment for affected patients.

Several recent studies have highlighted the promising diagnostic efficacy of blood-based biomarkers when assessing patients for PJI, including fibrinogen (FIB) levels[5], globulin (GLB) levels and the albumin to GLB (A/G) ratio [6], and the neutrophil to lymphocyte ratio (NLR) [7]. Yu, for example, reported NLR values to be superior to C-reactive protein (CRP) levels when diagnosing PJI with respect to their accuracy [7], while Yongyu Ye et al. determined GLB and A/G values to be promising biomarkers for PJI diagnosis [6]. Moreover, Lui et al. reported FIB values to exhibit sensitivity and specificity levels for the diagnosis of PJI similar to those of traditional biomarkers including CRP levels and the erythrocyte sedimentation rate (ESR) [5].

We have also previously reported the diagnostic utility of FIB levels in this context [8]. However, few studies to date have conducted detailed analyses of the diagnostic value of GLB levels, NLR, or A/G in PJI, leading us to conduct the present study in which we assessed the relative diagnostic value of these serological indices.

## Materials and methods

### Study design

This was a retrospective analysis of clinical data collected from patients diagnosed with either PJI or aseptic loosening from January 2017 – December 2020. Collected data included age, gender, and preoperative serum ESR, CRP, GLB, A/G, and NLR values. This study was approved by the Institutional Review Board.

### Inclusion criteria

Patients eligible for inclusion were individuals that had been diagnosed with chronic PJI after 90 days following TKA or THA or who had been diagnosed with aseptic loosening and underwent appropriate treatments in our department (revision arthroplasty or spacer insertion surgery) over the selected study period for whom preoperative CRP, ESR, GLB, A/G, and NLR values were available.

### Exclusion criteria

Patients were excluded from this study if they had been diagnosed with systemic inflammatory diseases (including inflammatory bowel disease, gout, sarcoidosis, multiple myeloma, lymphocytic leukemia, rheumatoid arthritis, psoriasis, polymyalgia rheumatica, hepatitis C/B infection, systemic lupus erythematosus, or myelodysplastic syndrome), exhibited tumors, were malnourished, had a history of trauma or dislocation within the past two weeks, or were missing key data.

### General patient information

In total, 194 patients were admitted to our hospital and diagnosed with PJI or aseptic loosening over the selected study period, of whom 115 met with the indicated inclusion and exclusion criteria. These patients were separated into PJI and aseptic loosening groups as appropriate, with the indicated demographic and clinical details discussed above being recorded for each patient.

### Clinical definitions

PJI was diagnosed as per the MSIS criteria(Table 1) [9].

Aseptic loosening was diagnosed using previously published criteria [10] based on a combination of pain in the hip/thigh region, knee pain, and radiological evidence consistent with loosening including displaced prosthesis components, a circumferential radiolucent line, or prosthesis component disintegration with the bone.

### Measurement approaches

Blood samples were collected on the morning following admission for all patients and used to measure CRP, GLB, ESR, A/G, and NLR values in the medical laboratory. The resultant data were collected from patient electronic medical records. The sensitivity and specificity of preoperative CRP, GLB, ESR, A/G, and NLR values when used for the diagnosis of PJI were assessed by comparing these values between the two groups of patients.

### Statistical analyses

Quantitative data are given as mean ± standard deviation and compared via Student’s t-tests or non-parametric tests as appropriate, with P < 0.05 as the significance threshold. Diagnostic performance was evaluated through receiver operating characteristics (ROC) analyses using MedCalc 19.0.4 (MedCalc Software, Ostend, Belgium), assessing parameters such as sensitivity, specificity, the diagnostic odds ratio (DOR), and the area under the ROC curve (AUC), with an AUC > 0.7 being considered to be acceptable. Youden’s index was used to define optimal thresholds for PJI diagnosis.

## Results

In total, the PJI and aseptic loosening groups included 53 and 62 patients, with no significant differences in demographic characteristics between these groups (Table 2).

**Table 1:** Definition of Periprosthetic Join Infection

PJI is diagnosed when patients meet one major criterion or 3/5 minor criteria	
Major Criteria	Two positive periprosthetic cultures with phenotypically identical organisms, OR A sinus tract communicating with the joint
Minor Criteria	1) Elevated serum CRP and ESR values; 2) Elevated synovial fluid white blood cell (WBC) counts OR + + changes on leukocyte esterase test strip; 3) An elevated polymorphonuclear neutrophil percentage (PMN%) in the synovial fluid; 4) Positive histological analyses of the periprosthetic tissue; 5) A single positive culture

The median GLB levels in the PJI and aseptic loosening groups were 31.700 g/L and 26.600 g/L, respectively, while the median A/G value in these groups was 1.150 and 1.510, respectively, the median NLR was 2.510 and 1.850, respectively, the median ESR was 53.000 mm/h and 16.000 mm/h, respectively, and the median CRP level was 24.890 mg/L and 2.245 mg/L, respectively (Table 3).

**Table 2:** Patient characteristics

Variable	PJI	aseptic loosening	P-value
Sex(Male)	24	32	0.592
Sex(Female)	29	30	
Age(median)	71.0(61–78)	68.5(59.5–74)	0.214

**Fig. 1:**
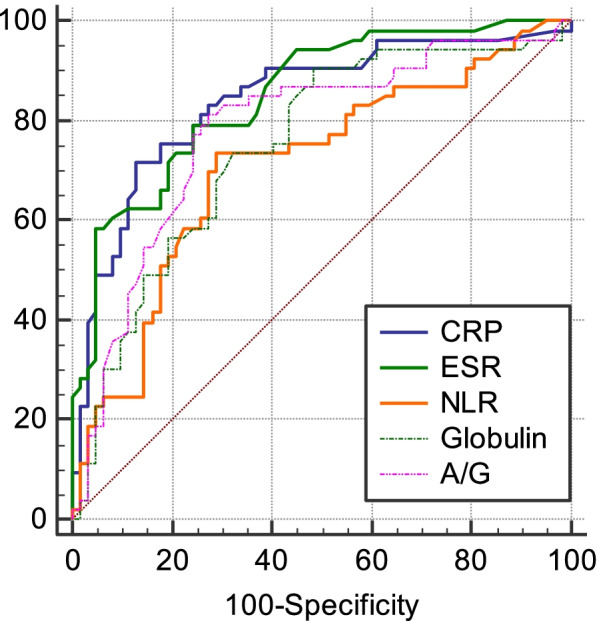
ROC curve analyses were used to gauge the relative value of C-reactive protein (CRP) levels, globulin levels, A/G, the erythrocyte sedimentation rate (ESR), and the neutrophil to lymphocyte ratio (NLR) as predictors of PJI

ROC curve analyses indicated that CRP exhibited the highest AUC value when used for the diagnosis of PJI (0.841), followed by the ESR (AUC = 0.850), NLR (AUC = 0.708), GLB levels (AUC = 0.747), and A/G (AUC = 0.779) (Fig. 1). At a cutoff value of 14.26 mg/ml, CRP exhibited relative sensitivity and specificity values of 71.7% and 87.1%. At a cutoff value of 32 mm/h, ESR exhibited respective sensitivity and specificity values of 79.25% and 75.81%. At a cutoff value of 2.1, NLR exhibited respective sensitivity and specificity values of 73.58% and 70.97%. At a cutoff of 26.6 g/L, GLB exhibited respective sensitivity and specificity values of 90.57% and 51.61%. At a cutoff value of 1.32, A/G exhibited respective sensitivity and specificity values of 81.13% and 72.58%.

GLB and A/G exhibited sensitivity values (90.57%, 81.13%) superior to those for CRP (71.70%) or ESR (79.25%), but corresponding specificity values (GLB: 51.61%, A/G: 72.58%) were significantly lower than those for CRP (87.10%) or ESR (75.81%). ROC analyses for NLR revealed that its sensitivity (73.58%) and specificity (70.97) did not offer significant advantages over corresponding values for CRP or ESR (Table 4).

**Table 3:** Median serological index values

Variable	PJI(*n* = 53)	aseptic loosening (*n* = 62)	*P*-value
Globulin	31.700 (28.400–35.300)	26.600 (24.375–30.550)	< 0.001
A/G	1.150 (0.960–1.255)	1.510 (1.265–1.670)	< 0.001
NLR	2.510 (1.900–3.335)	1.850 (1.425–2.362)	< 0.001
ESR	53.000 (35.000–76.500)	16.00 (7.000–33.000)	< 0.001
CRP	24.890 (10.595–54.095)	2.245 (0.8650-8.6075)	< 0.001

**Table 4:** The diagnostic performance of different serological parameters for PJI

	AUC	(95%CL)	Threshold	Sensitivity	Specificity
CRP	0.841	0.761–0.903	14.26 mg/L	71.70	87.10
ESR	0.850	0.771–0.910	32 mm/h	79.25	75.81
NLR	0.708	0.616–0.789	2.1	73.58	70.97
Globulin	0.747	0.658–0.824	26.6 g/L	90.57	51.61
A/G	0.779	0.692–0.851	1.32	81.13	72.58

## Discussion

The incidence of PJI occurs is driven by the development of a bacterial biofilm (BBF) attached to the implant surface. Initially, adherent bacteria generate a hydrated extracellular polysaccharide and protein matrix known as a glycocalyx [11]. The resultant biofilm serves as a barrier that can decrease antimicrobial agent ingress [12] such that antibiotics commonly only eliminate a subset of the bacteria present therein, resulting in the transient alleviation of symptoms followed by subsequent re-infection when additional bacteria are released from the BBF [11]. These biofilms hamper efforts to treat patients affected by chronic PJI, which is often difficult to diagnose owing to its atypical symptoms and insidious onset. Several blood-based biomarkers have been identified in recent years as being of value for the diagnosis of PJI, including soluble intercellular adhesion molecule-1 (sICAM-1), myeloid-related protein 14 (MRP-14), soluble urokinase plasminogen activation receptor (su-PAR), and lipopolysaccharide-binding protein (LBP). Despite promising reports regarding their performance in the context of PJI diagnosis, these markers require specialized antibodies and entail higher costs that make them difficult to routinely measure in clinical practice [8]. There is thus a clear need to develop more convenient and efficient tests for the diagnosis of PJI.

While CRP, ESR, and WBC are markers that are routinely used to diagnose infections, they lack sensitivity or specificity when diagnosing PJI. Bedair et al. [13] reported that serum CRP exhibits just 53% sensitivity when diagnosing PJI. As such, several other research teams have explored alternative serum biomarkers for PJI. Alisina et al., for example, highlighted the promise of serum D-dimer levels in the diagnosis of PJI when planning optimal reimplantation timing, with D-dimer being included in PJI diagnostic guidelines [14]. Despite such promise, however, serum D-dimer levels do not offer advantages over ESR or CRP when used to diagnose PJI [15].

Angkananard et al. previously reported NLR to offer value as a predictor of infected patient outcomes [16], while Meyer et al. determined that A/G ratio values are correlated with infection status [17], and Schmilovitz-Weiss et al. reported the A/G ratio to predict cancer patient outcomes [18]. These findings have led many researchers to explore the relationships among GLB, NLR, A/G, and PJI. In their report, Yu et al. found NLR values to be more accurate than CRP levels when diagnosing early PJI. In a separate study, Ye et al. found both GLB and the A/G ratio to offer promise as adjuvant biomarkers when diagnosing PJI.

In the previous report published by Yu et al. ROC curve analyses for NLR suggested it to exhibit promising diagnostic utility. However, our ROC curve analyses, when conducted using the calculated cutoff value, did not exhibit similar promising results, as reported previously [19]. While NLR offered some value as a diagnostic tool for PJI detection, it performed with lesser diagnostic efficacy than that observed for ESR or CRP levels. There are several possible reasons for these findings. For one, the study conducted by Yu et al. primarily included patients with early-stage PJI, whereas we herein focused on individuals with chronic PJI. Given that these patients are more common in our clinic, these results may be of greater clinical significance. We additionally found that GLB and A/G values may offer value when diagnosing PJI, but that their diagnostic performance does not exceed that for CRP or ESR. In addition, other factors have the potential to influence specificity including ankylosing spondylitis, rheumatoid arthritis, tuberculosis, and hematopoietic failure, potentially limiting the value of these serological indices in real-world clinical contexts.

## Limitations

This study is subject to certain limitations. For one, only 115 patients were included in this study. Secondly, patients with acute PJI, who account for only a small fraction of the overall patients in our department, were excluded from this study.

## Conclusions

Our results suggest that globulin levels, NLR values, and A/G values do not offer significant advantages over ESR or CRP values when employed for the diagnosis of PJI. However, additional large-scale studies will be essential to confirm and expand upon these results.

## Supplementary Information

**Additional file 1.** (XLSX 21 kb)

## Data Availability

The data that support the findings of this study are available on request from the corresponding author.

## Abbreviations

CRP: C-reactive protein
ESR: Erythrocyte Sedimentation Rate
GLB: Globulin
A/G: Albumin to Globulin Ratio
NLR: Neutrophil to Lymphocyte Rate
PJI: Periprosthetic Joint Infection
FIB: Fibrinogen
